# Exploring the role of Cdk5 on striatal synaptic plasticity in a 3-NP-induced model of early stages of Huntington’s disease

**DOI:** 10.3389/fnmol.2024.1362365

**Published:** 2024-11-06

**Authors:** Elizabeth Hernández-Echeagaray, Jorge A. Miranda-Barrientos, Elizabeth Nieto-Mendoza, Francisco Miguel Torres-Cruz

**Affiliations:** ^1^Laboratorio de Neurofisiología del Desarrollo y la Neurodegeneración, Unidad de Biomedicina, FES-I, Universidad Nacional Autónoma de México, Mexico City, Mexico; ^2^Lieber Institute for Brain Development, Baltimore, MD, United States

**Keywords:** Cdk5, neuroprotection, plasticity, DARPP-32, 3-NP

## Abstract

Impaired mitochondrial function has been associated with the onset of neurodegenerative diseases. Specifically, certain mitochondrial toxins, such as 3-nitropropionic acid (3-NP), initiate cellular changes within the striatum that closely resemble the pathology observed in Huntington’s disease (HD). Among the pivotal signaling molecules contributing to neurodegeneration, cyclin-dependent kinase 5 (Cdk5) stands out. In particular, Cdk5 has been implicated not only in cellular pathology but also in the modulation of synaptic plasticity. Given its widespread presence in the striatum, this study seeks to elucidate the potential role of Cdk5 in the induction of corticostriatal synaptic plasticity in murine striatal cells subjected to subchronic doses of 3-NP *in vivo*, aiming to mimic the early stages of HD. Immunostaining analyses revealed an increase in Cdk5 in tissues from animals treated with 3-NP, without a significant change in protein levels. Regarding striatal plasticity, long-term depression (LTD) was induced in both control and 3-NP cells when recorded in voltage clamp mode. The Cdk5 inhibitor roscovitine-reduced LTD in most cells. A minority subset of cells exhibited long-term potentiation (LTP) generation in the presence of roscovitine. The inhibitor of D1 receptors SCH23390 prevented LTP in three of nine cells, implying that MSN cells lacking D1/PKA activation were capable of LTP induction when Cdk5 was also blocked. Nevertheless, the co-administration of H89, a PKA inhibitor, along with roscovitine, prevented the generation of any type of plasticity in all recorded cells. These findings show the impact of 3-NP treatment on striatal plasticity and suggest that Cdk5 during early neurodegeneration may attenuate signaling pathways that lead neurons to increase their activity.

## 1 Introduction

Mitochondrial dysfunction plays a pivotal role in inducing synaptic alterations and cellular dysfunction observed in various neurodegenerative diseases (Johri and Beal, 2012; Gillingwater and Wishart, 2013). One specific mitochondrial toxin known for its irreversible inhibition of succinate dehydrogenase (SDH) within complex II is 3-nitropropionic acid (3-NP). Due to its ability to induce striatal excitotoxicity, degeneration, synaptic impairments, and neuronal death, resembling those found in Huntington’s disease (HD), 3-NP has been widely utilized as a model for HD research (Beal et al., 1993; Johnson et al., 2000; Cakała et al., 2006; Saulle et al., 2004; Brouillet et al., 2005; Hernández Echeagaray et al., 2012).

Furthermore, several studies have documented that both *in vitro* and *in vivo* administration of 3-NP leads to aberrations in synaptic plasticity, including impaired long-term depression (LTD), abnormal induction of long-term potentiation (LTP), and failure in depotentiation (Calabresi et al., 2001; Dalbem et al., 2005; Picconi et al., 2006). The precise molecular mechanisms underlying these plasticity abnormalities remain elusive, although alterations in the expression levels of several molecules crucial for normal synaptic plasticity in the striatum have been reported following 3-NP treatment (Napolitano et al., 2004). Notably, among these molecules, Cdk5 has been identified as one whose expression is affected by 3-NP (Napolitano et al., 2004). In addition, in striatal cells from knock-in mice with mutant huntingtin, Cdk5 levels are decreased but its phosphorylation is increased (Paoletti et al., 2008).

Cdk5, a serine/threonine kinase prominently expressed in the striatum, becomes activated through binding with its activator proteins p35 or p39, forming an active complex (Ishiguro et al., 1994; Lew et al., 1994; Tsai et al., 1994). Known for playing a crucial role in various physiological processes, including synaptic plasticity and learning (Angelo et al., 2006; Hawasli and Bibb, 2007; Posada-Duque et al., 2017; Liao et al., 2021), Cdk5 has been the subject of our previous findings, revealing that inhibition of Cdk5 activity promotes striatal LTP (Miranda-Barrientos et al., 2014). Furthermore, existing literature indicates an increase in Cdk5 expression (Napolitano et al., 2004) or activation in the striatum of animal models exhibiting Huntington’s disease (HD), where synaptic plasticity alterations have been documented (Crespo-Biel et al., 2007).

This study evaluates whether Cdk5 participates in corticostriatal synaptic plasticity within the dorsal striatal tissue of mice treated *in vivo* with low, subchronic doses of 3-NP. This approach mimics the early stages of HD (Rodríguez et al., 2010; Hernández Echeagaray et al., 2012).

## 2 Materials and methods

This study used 5-week-old male C57BL/6 mice; 6 for histology, 10 for Western blotting (WB), and 32 for electrophysiology. Upon arrival, the mice were randomly assigned to either the control vehicle group or the 3-NP-treated group. They were then group-housed in plastic boxes, with five animals per cage, ensuring free access to food and water. The housing conditions included a 12/12-h dark/light cycle, and the mice were maintained at a room temperature of 24–25°C. All animal care and handling procedures were conducted in accordance with the Official Mexican Norm (NOM-062-ZOO-1999) for the care and use of experimental animals and were approved by our Institutional Bioethics Committee. Efforts were made to minimize the number of mice used.

### 2.1 3-NP treatment

The 3-NP was solubilized in phosphate buffer (PB) and pH-adjusted to 7.4. Intraperitoneal (i.p.) injection of 3-NP crosses the blood–brain barrier and mimics the neuropathology of HD (Hamilton and Gould, 1987; Borlongan et al., 1997, Rodríguez et al., 2010). The treatment protocol involved daily i.p. injections of 3-NP at a dosage of 15 mg/kg for a period of 5 days. Control mice, in contrast, received only PB (i.p.) throughout the treatment duration. All experiments were conducted 2 days following the administration of the last treatment injection.

### 2.2 Obtaining sections and electrophysiology

Mice were anesthetized using halothane in a drop-in induction chamber and euthanized by decapitation. The brain was swiftly extracted and immersed in ice-cold (4°C) low-calcium saline, comprising 130 mM NaCl, 3 mM KCl, 5 mM MgCl_2_, 26 mM NaHCO_3_, 1.25 mM NaH_2_PO_4_, 1 mM CaCl_2_, and 10 mM glucose, to protect from excitotoxicity-induced damage during the cutting process. Sagittal brain slices (300 μm) were obtained using a vibratome (PELCO, 1000 Plus Sectioning System, Ted Pella Inc., Redding, CA, United States). These sections were maintained in the same low-calcium saline solution and continuously oxygenated (95% O_2_, 5% CO_2_) for 1 h before electrophysiological experiments.

Following the incubation period, the slices were transferred to a recording chamber continually perfused with recording saline (130 mM NaCl, 3 mM KCl, 2 mM MgCl_2._, 26 mM NaHCO_3_, 1.25 mM NaH_2_PO_4_, 2 mM CaCl_2_, and 10 mM glucose) and consistently bubbled with 95% O_2_/5% CO_2_. Borosilicate electrodes (3–5 MΩ) filled with an internal solution containing 72 mM KH_2_PO_4_, 36 mM KCl, 2 mM MgCl_2_•6H_2_O, 10 mM HEPES sodium salt, 1.1 mM EGTA, 0.2 mM Na_2_ATP, 0.2 mM Na_3_GTP, and 5 mM QX-314 were used for whole-cell voltage clamp recordings. QX-314 was present in the internal solution to inhibit voltage-gated sodium channels and avoid the influence of neuronal firing.

Medium spiny neurons (MSN) were visualized using infrared differential interference contrast (DIC) illumination on an upright microscope (BX51WI, Olympus) equipped with water immersion objective magnification (Olympus X Lum Plan Fl 20x/0.95 W, Japan) coupled to a CCD camera (charge-coupled-device, Hitachi) and identified by their membrane properties (input resistance, membrane capacitance, and time constant, see Table 1). Bicuculline (10 μM) was present in all experiments to study corticostriatal excitatory postsynaptic currents (EPSCs). In experiments aimed at assessing the role of Cdk5 in plasticity, the inhibitor roscovitine (20 μM) was applied via bath application before stimulation. For experiments targeting D1 receptors, the antagonist SCH23390 (1 μM) was applied before roscovitine. Similarly, experiments focusing on PKA involved the bath application of the inhibitor H89 (5 μM) prior to roscovitine application. Electrical stimulation was conducted in the corpus callosum using a tungsten bipolar electrode coupled to a constant voltage stimulator isolator (DS2A-MK. II, Digitimer Ltd., Hertfordshire, United Kingdom). To elicit EPSC responses, a stimulus ranging from 10 to 20 V, with a duration of 50 to 100 μs and a frequency of 0.1 Hz, was applied using an isolated stimulator. The stimulus intensity was calibrated to 50% of the maximum amplitude of the EPSC response. In all cases, a paired-pulse stimulation consisting of two stimuli (S_1_ and S_2_) of the same duration (100 μs) and intensity (previously calibrated), separated by an interval of 30 to 50 ms at 0.1 Hz, was delivered through the bipolar electrode (See Supplementary Figure S1).

**Table 1:** Membrane properties.

Control (*n* = 7)	Mean	SD ±	SE ±
Cm	162.04	14.15	5.35
RN MΩ	70.12	17.36	6.56
Ra MΩ	27.82	2.12	0.80
τ (ms)	3.73	0.46	0.17

3-NP (*n* = 38)	Mean	SD ±	SE ±
Cm	162.39	34.14	5.94
RN MΩ	76.38	35.68	6.21
Ra MΩ	27.52	10.28	1.78
τ (ms)	3.52	1.10	0.19

Recorded EPSCs from MSNs were amplified and digitized using an Axopatch 200B amplifier coupled to a Digidata 1322A digitizer (Axon, Molecular Devices Corp.CA, United States). Throughout the experiment, input and access resistance were continuously monitored by evoking a transmembrane current with a voltage command (10 mV) in a whole-cell voltage clamp. Time constants were determined after whole-cell compensation, and capacitance was measured using the Axopatch dial. The access resistance (RN) was determined by fitting the I-V relation with a polynomial function. Throughout each experiment, monitoring was facilitated through the membrane test function of pClamp software at −70 mV, ~100 Hz acquisition rate, and 1 Hz filtering. Acceptance criteria included an access resistance below 30 MΩ, consistent across all experiments. Voltage holding was maintained at −70 mV.

For synaptic plasticity induction, high-frequency stimulation (HFS) consisting of three trains of 100 Hz, lasting 3 s each, with intervals of 20 s between each train, was administered. The amplitudes of EPSCs before and after the trains were analyzed in the last 10 min before the train and after the train during the last 10 min of the time course.

### 2.3 Tissue preparation for free-floating sections immunohistochemistry

Mice were deeply anesthetized with halothane prior to transcardial perfusion as described above. The perfusion process involved initially flushing the circulatory system with a saline solution (NaCl 0.15 M), followed by perfusion with phosphate-buffered solution (PB 0.1 M and pH 7.4) containing 4% paraformaldehyde (PFA) in 0.15 M PB at pH 7.4. This procedure was carried out at 4°C to ensure optimal preservation of tissue integrity.

Following the removal from the skull, the brains were postfixed in the same fixative at room temperature (RT) for 2 h. Subsequently, they were transferred to a PB-sucrose solution (30%) to facilitate cryopreservation. The brains were then frozen in cold 2-methylbutane and cut into 30-μm-thick coronal sections using a cryostat. These sections were systematically collected into 24-well dishes filled with a blocking solution, preparing them for subsequent immunohistochemistry analyses (3% bovine serum albumin, 0.1% Triton X-100, and 0.025% sodium azide in PB).

The brain sections underwent an overnight incubation in a blocking serum at 4°C. Following this, the tissue slices were treated with a primary antibody targeting Cdk5 (1:500; Santa Cruz Biotechnology, Inc.), appropriately diluted in the blocking solution, and incubated for 24 h at 4°C. Subsequently, three 15-min washes were conducted using the blocking solution to ensure proper antibody removal. The tissue slices were then subjected to a 90-min incubation at room temperature with the corresponding biotinylated secondary antibody (Vector Laboratories, Burlingame, CA, United States) diluted (1:500) in the blocking solution.

Following three washes with PB, the slices underwent incubation using an avidin–peroxidase kit (VECTASTAIN® ABC-HRP, Peroxidase Staining Kit, Vector). Peroxidase activity was subsequently revealed and intensified using 3,3′-diaminobenzidine as a chromogen and nickel chloride as outlined in the supplier’s protocol. The staining process was halted after approximately 5 min by thoroughly washing the slices with PB. The treated slices were then carefully mounted on gelatin-coated slides, left to air-dry overnight, and cover-slipped with Permount (Fisher Chemical™). Specific immunostaining was detected as a diffuse brown precipitate.

### 2.4 Analysis of stainings

To assess the quantity of Cdk5-positive neurons, brain serial coronal sections from six mice (three mice per experimental group) containing the striatum were digitized (4X, 10X, and 40X). These digitized images were captured using a light microscopy setup adapted to an image analysis system (Universal Imaging, Motic). Subsequently, the images were utilized to document the expression of Cdk5 in the striatum and subjected to analysis through ImageJ software provided by the NIH. Then, an unbiased counting frame of 2 mm^2^ per field (10X) was generated, and the frames were obtained from the dorsal striatum (one frame per slice and nine slices per mouse brain), enabling the counting of stained cells. The brightness, contrast, and threshold settings remained unchanged, ensuring consistency, while the particle analyzer of ImageJ software was employed to accurately detect the cells. Following the comprehensive counting of all cells within the sampling frame, the total number of cells was estimated. Statistical analysis of cell counts was performed using Student’s *t*-test, and significance was established at *p* < 0.05.

### 2.5 Immunoblotting

To evaluate the phosphorylation status of DARPP-32 at Thr-75, DARPP-32 at Thr-34, and total DARPP-32, striatal slices from animals treated with either 3-NP (*n* = 5 mice) or vehicle control (*n* = 5 mice) were incubated in the physiological saline solution used for electrophysiological experiments, within an 8-well Petri dish that was continuously oxygenated (95% O_2_ and 5% CO_2_). This incubation included the addition of bicuculline (10 μM), roscovitine (20 μM), or a combination of roscovitine (20 μM) and nifedipine (5 μM) for 20 min. Following incubation, the slices were homogenized in a lysis buffer comprising 26 mM Tris–HCl, 1% Triton X-100, 1.3 M glycerol, 130 mM NaCl, and a protein phosphatase inhibitor cocktail (Complete™ mini-tab, Roche). To evaluate Cdk5, protein-level striatal tissue was processed as described above, but without the reagents tested in electrophysiological experiments.

Following collection, the samples were subjected to centrifugation for 5 min at 4,500 rpm, and the resultant supernatant was collected and stored at −70°C for subsequent analysis. Protein quantification was conducted using the Bradford method.

In the subsequent blotting procedure, 30 μg of protein was loaded onto a 10% polyacrylamide electrophoresis gel. The proteins were then transferred to PVDF transfer membranes, blocked with 10% skinny non-fat dry milk dissolved in TBS (pH 7.6) for 1 h and incubated with primary antibodies against phospho-DARPP-32^Trh-34^ (GeneTex, Cat. No. GTX55025, 1:2,000), phospho-DARPP-32^Trh-75^ (Cell Signaling Technology, Cat. No. 2301, 1:1,000), DARPP-32 (GeneTex, Cat. No. GTX82715, 1:1,000), Cdk5 (sc-249, Santa Cruz, 1:200–1:1,000), and *β*-actin (GeneTex, Cat. No. GTX629630, 1:60000–1:120000) as a load control for 12 h at 4°C. Secondary antibodies (Anti-rabbit IgG, HRP, Cell Signaling Technology, Cat. No. 7074, 1:2000–1:10000) were then applied for 2 h at room temperature. All antibodies were diluted in TBS. The protein level of Cdk5 was detected using chemiluminescence and scanned with an AC-Digit Western Blot scanner. The intensity of immune-reactive bands was analyzed using Image Studio Digits 4.0 software, while DARPP-32 was identified utilizing the chemiluminescence method with Immobilon™ Western Chemiluminescent HRP Substrate from Millipore (Billerica, MA, United States). Subsequently, images were captured using the Gel Documentation System from BioSens SC 645, a product of Shanghai Bio-Tech Co., Ltd. The relative protein level was determined by comparing the optical density of Cdk5 to the expression of *β*-actin on the same membrane. This ratio was then normalized to the control expression. Additionally, for DARPP-32, the ratio of phospho-DARPP-32/total DARPP-32 level was assessed and similarly normalized to control expression.

### 2.6 Drugs

All reagents were purchased from Sigma-Aldrich Merck (Darmstadt, GER) unless otherwise stated.

### 2.7 Statistical analysis

A one-way ANOVA with a *post-hoc* test or Student’s *t*-test (paired or unpaired, depending on the experiment) was used for data comparisons for WB analyses and between pre-HFS and post-HFS in recorded neurons. Non-normal distribution data were analyzed using a non-parametric test. Statistically significant differences were considered at *p* < 0.05.

## 3 Results

### 3.1 3-NP treatment increases Cdk5 immunostaining

**Figure 1:**
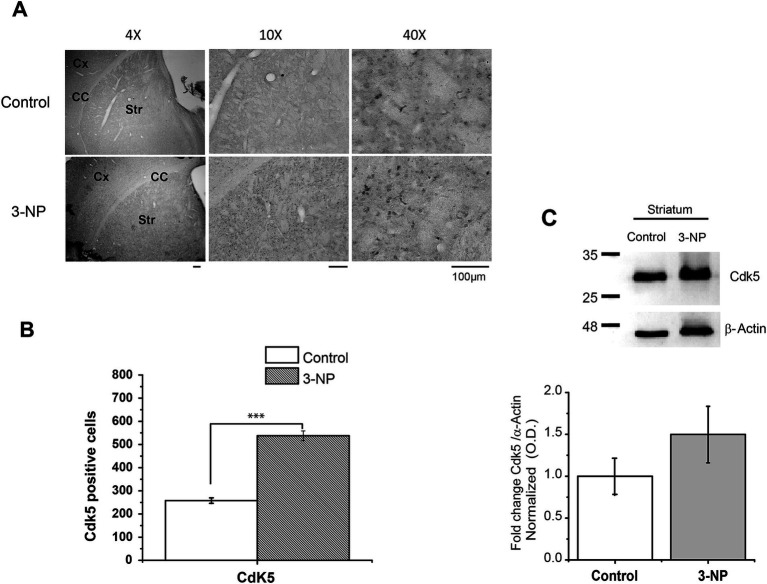
Immunohistochemical and immunoblot expression of Cdk5 in striatal tissue of 3-NP-treated mice. **(A)** Immunostaining of Cdk5 in a coronal section (4X, 10X, and 40X) from the striatum of control and 3-NP-treated mice, respectively. Note that Cdk5 is present in the soma of the striatal cells. Cx (cerebral cortex), CC (corpus callosum), and Str (striatum). Scale bar: 100 μm. **(B)** Counts of Cdk5-positive stained cells. 3-NP treatment *in vivo* significantly increased Cdk5 immunostaining after 5 days of treatment (15 mg/kg, i.p.) (control = 257 ± 11.99 vs. 3-NP = 537.630 ± 20.87, *t*_52_ = −11.61, ***p < 0.001). **(C)** Cdk5 protein level present a non-significant increase in striatal tissue of 3-NP-treated animals (control = 1.00 ± 0.25 vs.3-NP = 1.49 ± 0.337, *t_4_* = −0.997, *p* = 0.375; *n* = 5 per group).

First, we analyzed whether 3-NP produced changes in Cdk5 staining and protein-level expression. The results showed that Cdk5 immunolocalization significantly increased the number of Cdk5-positive striatal cells from 3-NP-treated mice compared to that in control mice (Figures 1A,B). However, the protein level of Cdk5 in the striatum exhibited a non-significant increase compared to the control striatal tissue (Figure 1C).

### 3.2 3-NP treatment alters striatal plasticity

**Figure 2:**
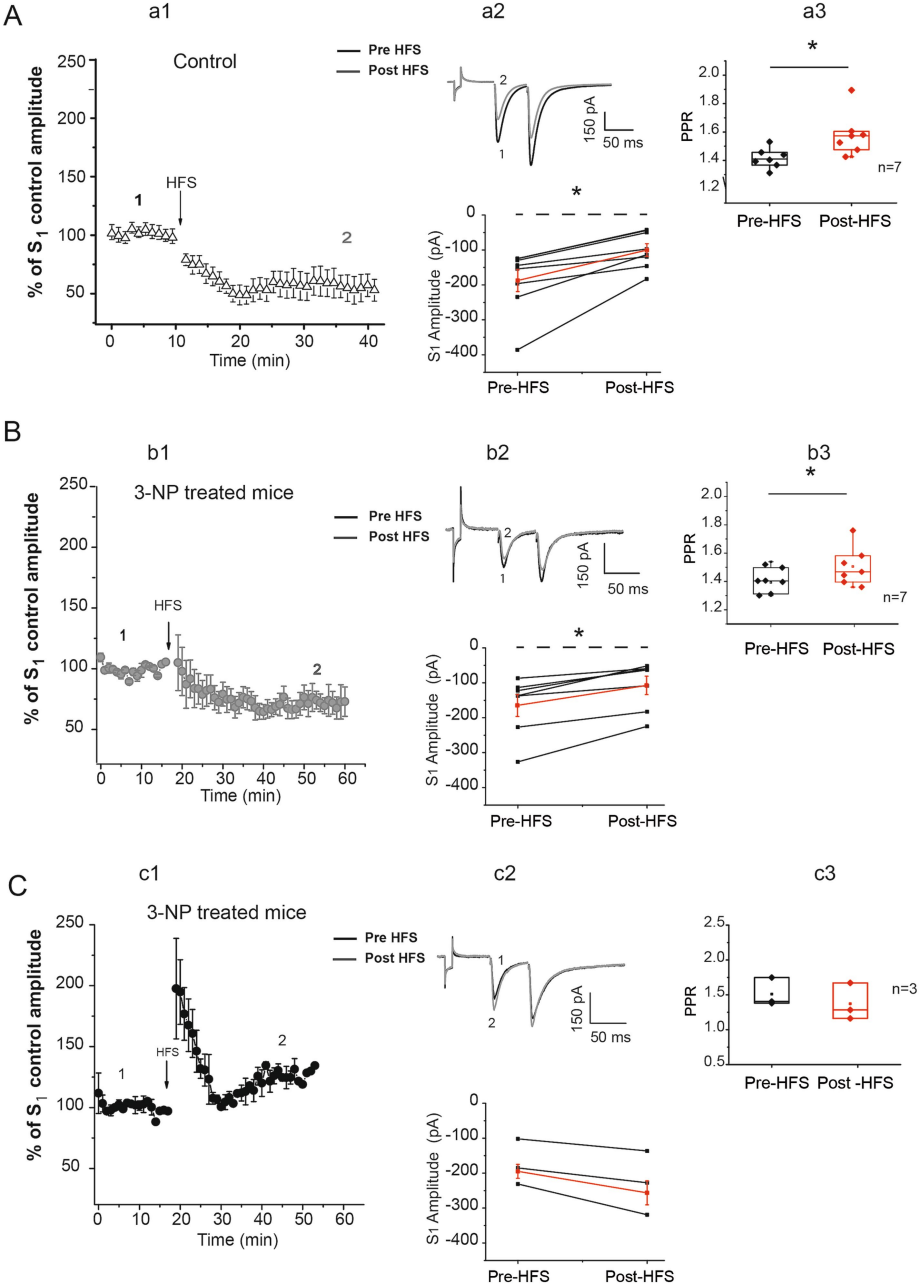
3-NP treatment alters striatal plasticity. **(A)** Time course of S_1_ normalized to control, obtained from control slices (a1). After HFS, a robust LTD was generated in 7 of 7 cells from 7 mice (pre-HFS amplitude average = −186.74 ± 31.56 pA vs. post-HFS amplitude average = −99.51 ± 18.19 pA; *t_6 =_* − 4.566; *p* < 0.002, two-tailed paired *t*-test, a2 bottom). Representative traces before (1) and after HFS (2) are illustrated (a2 top). PPR was significantly affected in striatal LTD after HFS (pre-HFS median = 1.44 vs. post-HFS median = 1.57; *W* = 28, *p* = 0.022, Wilcoxon *t-*test; a3). **(B)** Time course of S_1_ normalized to control, obtained in slices from 3-NP-treated mice (b1). LTD was generated in 7 of 10 cells from slices of seven 3-NP-treated mice (gray circles). After HFS train, LTD was observed (pre-HFS amplitude average = −164.301 ± 31.599 pA vs. post-HFS amplitude average = −107.044 ± 26.346 pA; *t*_6_ = −5.426, *p* < 0.001, two-tailed paired *t*-test, in this figure b2 bottom). Representative traces of pre-HFS (1) and post-HFS (2) are shown (b2 top). Note LTD generated of 3-NP-treated mice slices was smaller than that in control mice. The PPR was significantly affected (pre-HFS median = 1.40 vs. post-HFS median = 1.45; W = 28, *p* = 0.016 Wilcoxon *t-*test; b3). **(C)** Time course of cells presenting LTP in slices from 3-NP-treated mice; 3 of 10 neurons exhibited a non-significant amplitude potentiation after HFS (pre-HFS = −172.72 ± 37.875 pA vs. post-HFS ^=^ − 227.67 ± 52.65 pA; *t*_2_ = 3.316, *p* = 0.080, two-tailed paired *t*-test). PPR did not exhibit differences (pre-HFS median = 1.404 vs. post-HFS median = 1.283, W = −6, *p* = 0.181, Wilcoxon *t-*test; 2c3).

Synaptic plasticity impairments have been reported in different models of HD and striatal degeneration. To test whether 3-NP administration *in vivo*-induced synaptic plasticity alterations in our striatal degeneration model, patch-clamp recordings from corticostriatal connections were carried out. In brain slices from seven control mice, HFS produced a significant LTD in the EPSC amplitude of all 7 medium spiny neurons (MSNs) after HFS training (Figure 2A, a1,a2). The analysis of the paired-pulse ratio (PPR) before and after HFS in control tissue, was significantly different in the PPR ratio suggestive of presynaptic mechanisms underlying LTD generation (Figure 2A, a3).

The analysis of plasticity induced in the neurons of seven 3-NP-treated mice exhibited two types of plasticity; 7 of 10 cells exhibited LTD after HFS (Figure 2B, b1). The PPR after HFS was significantly different indicating presynaptic mechanisms (Figure 2B, b3). Furthermore, the LTD observed in slices from 3-NP-treated mice was significantly smaller than that observed in control mice. Three neurons out of 10 exhibited LTP after HFS (Figure 2C), with a PPR that did not exhibit statistical differences after HFS, suggesting that postsynaptic mechanisms are involved in this LTP generation (Figure 2C, c3).

### 3.3 Inhibition of Cdk5 in 3-NP-treated animals affected striatal plasticity

**Figure 3:**
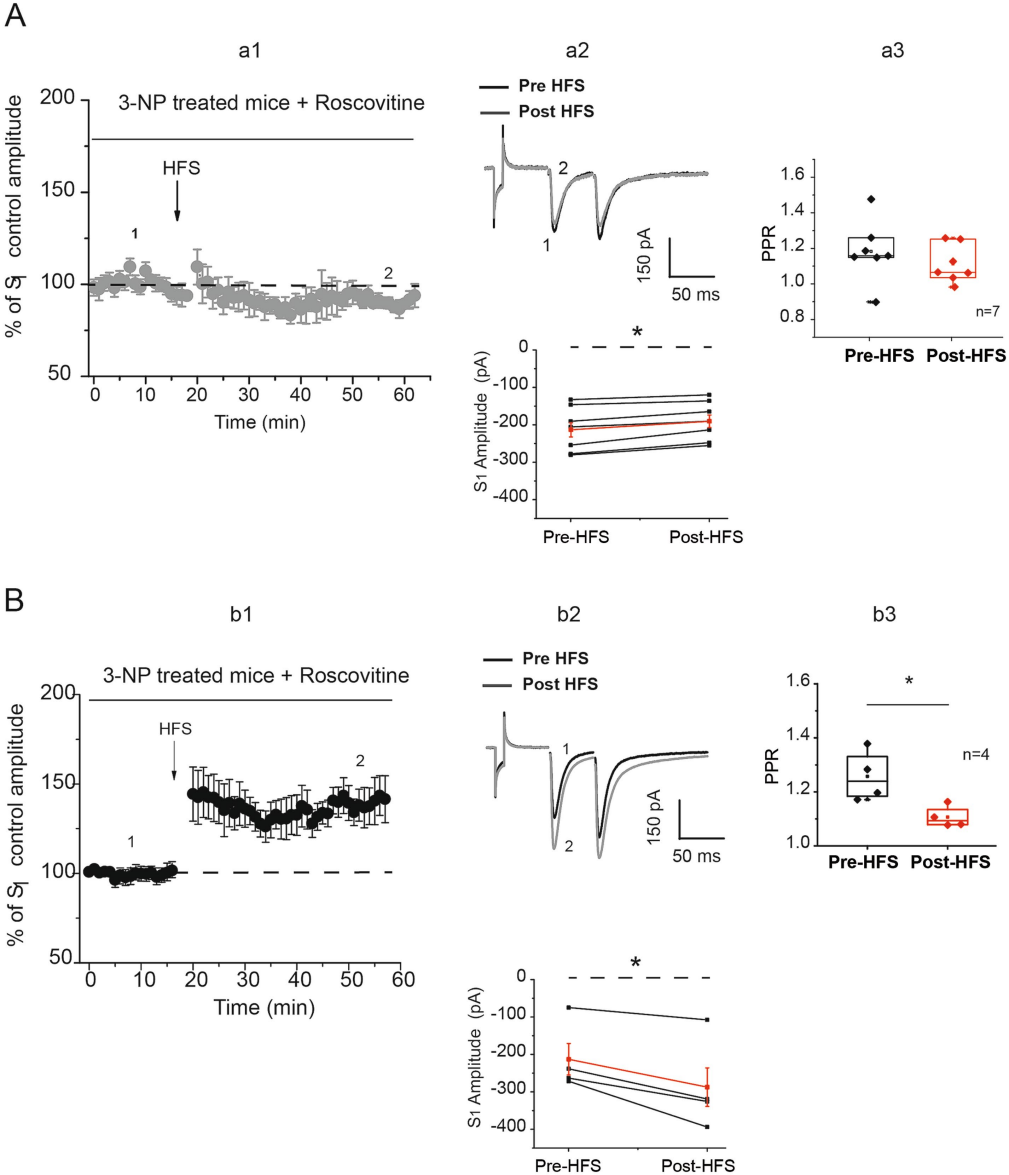
Cdk5 inhibition modifies striatal plasticity. **(A)** Time course of S_1_ normalized to control, recorded in 7 of 11 neurons from seven 3-NP-treated mice that generate discrete but significant LTD after HFS (gray circles a1) in the presence of 20 μM of roscovitine (pre-HFS = −212.439 ± 22.817 pA vs. post-HFS = −189.63 ± 19.87 pA, *t*_6_ = −5.553, *p* < 0.001, two-tailed, paired *t*-test, a1, a2 bottom). Traces before (1) and after HFS (2) are illustrated (a2 top). PPR of LTD induced in 3-NP recorded neurons was not affected after HFS (pre-HFS = 1.17 ± 0.062 vs. post-HFS =1.107 ± 0.0391, *t_6_* = 1.843, *p* = 0.115, two-tailed, paired *t*-test, a3). **(B)** Time course S_1_ normalized to control recorded in neurons (4 of 11) from seven 3-NP-treated mice in the presence of roscovitine (b1). LTP was generated after HFS (pre-HFS amplitude = −212.00 ± 46.28 pA vs. post-HFS amplitude = −286.50 ± 61.95 pA, *t*_3_ = 4.003, *p* = < 0.028, two-tailed, paired *t*-test, black circles, b2 bottom). Representative traces of pre-HFS (1) and post-HFS (2) are shown (b2 top). The PPR of LTP was significantly affected after HFS (pre-HFS = 1.25 ± 0.046 vs. post-HFS = 1.10 ± 0.019, *t*_3_ = 4.344, *p* = 0.02, two-tailed, paired *t*-test).

In our previous study, we established a correlation between Cdk5 activity and synaptic plasticity in the striatum. To determine whether Cdk5 plays a role in corticostriatal synaptic plasticity in slices from mice treated with 3-NP, we selectively inhibited its activity using the Cdk5 inhibitor roscovitine (20 μM). Under the influence of roscovitine, corticostriatal synapses of neurons from 3-NP-treated mice exhibited LTD and LTP (Figure 3A, a1,a2). Seven out of 11 cells from 3-NP-treated mice exhibited LTD in the presence of roscovitine after HFS (Figure 3A, a1,a2). This LTD is discrete in comparison with that observed in neurons from control mice or 3-NP-treated mice without roscovitine. The PPR analysis demonstrated that LTD induced under these conditions was not caused by presynaptic mechanisms (Figure 3A, a3). Few cells from the 3-NP-treated mice generated LTP (4/11) in the presence of roscovitine after HFS. The PPR from these experiments was statistically different after HFS (Figure 3B, b3).

### 3.4 The PKA signaling pathway is involved in roscovitine plasticity induction in 3-NP animals

**Figure 4:**
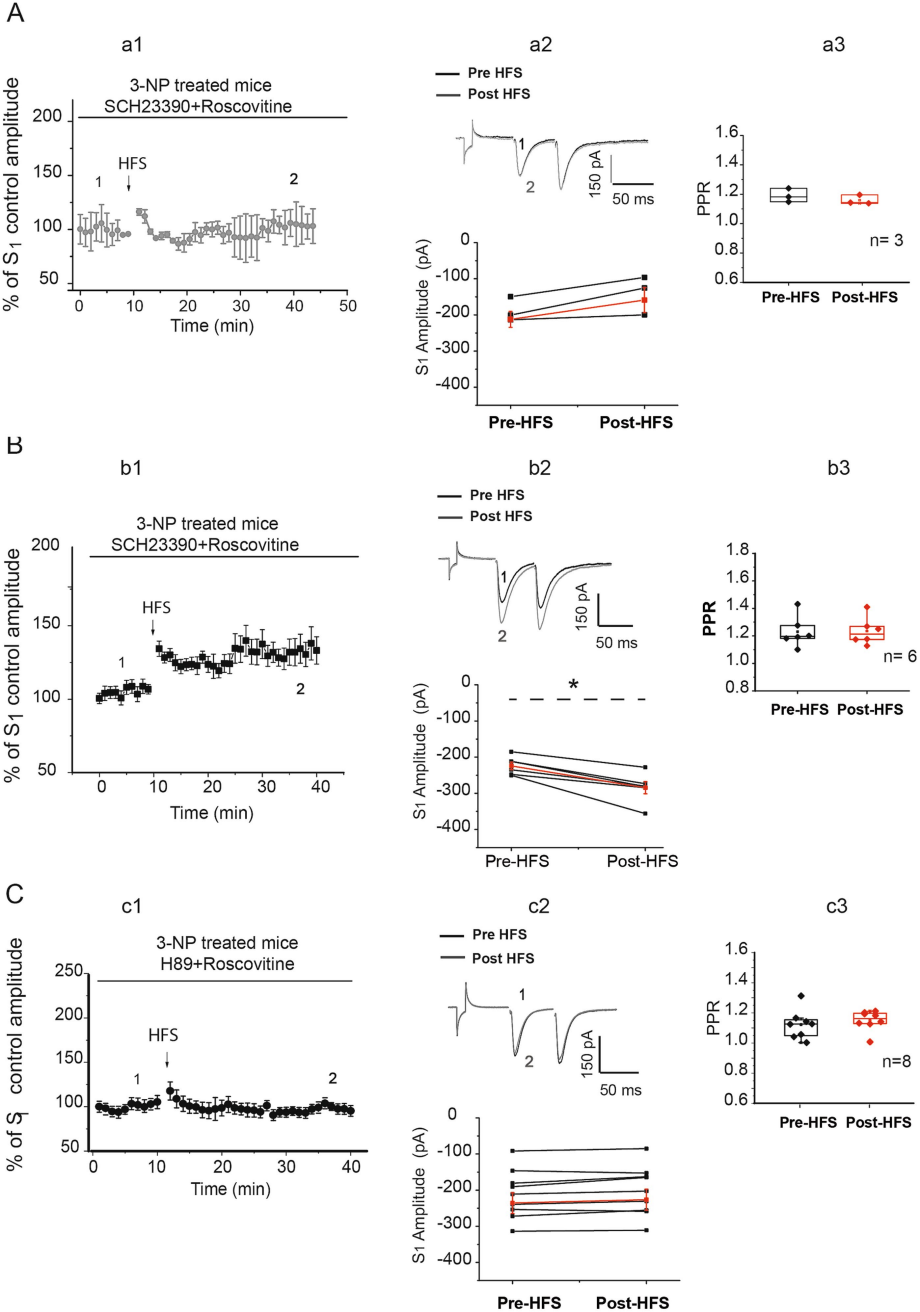
PKA role in striatal plasticity changes induced by Cdk5 inhibition in neurons from 3-NP-treated mice. **(A)** Time course of the effect of 1 μM SCH23390 on EPSC in the presence of roscovitine. Data are responses to S1 normalized to control (a1). The D1 receptor antagonist SCH 23390 prevented the development of plasticity in three of nine cells from seven mice (pre-HFS amplitude = −187.81 ± 19.46 pA, vs. post-HFS amplitude = −140.39 ± 30.83 pA, *t*_2_ = −2.650, *p* = 0. 118, two-tailed *t*-test; a2 bottom). Traces before (1) and after HFS (2) from cells that did not display plasticity are shown in a2 top. PPR did not show differences before (1. 190 ± 0.026) and after HFS (1.159 ± 0.018, *t_2_* = 2.890, *p* = 0.102, two-tailed, paired *t*-test, a3). **(B)** Time course of the S_1_ normalized to control in the presence of SCH23390 1 μM + roscovitine 20 μM (b1). In 6 of 9 cells from 7 mice, SCH23390 did not block LTP after HFS (pre-HFS = −223.57 ± 10.26 pA, vs. post-HFS = −283.90 ± 16.76 pA, *t*_5_ = 5.833, *p* = 0.002, two-tailed, paired *t*-test; black circles, b2 bottom). Representative traces before (1) and after HFS (2) are shown (b2, top). The PPR of LTP produced in the presence of SCH 23390 + roscovitine was not affected after the HFS (pre-HFS median = 1.196 vs. post-HFS median = 1.214, W = 1, *P* =, Wilcoxon *t*-test, b3). **(C)** Time course of EPSC recorded in neurons from 3-NP-treated mice in the presence of the PKA blocker H89 5 μM + roscovitine 20 μM, (c1). H89 impedes the development of plasticity in eight/eight recorded cells (pre-HFS = −210.867 ± 25.579 pA vs. post-HFS = −202.55 ± 25.95 pA, *t*_7_ = −2.072, *p* = 0.07, two-tailed, paired *t*-test, c2 bottom). Representative traces from cell recordings from striatal slices obtained before (black traces) and after HFS (gray traces) are illustrated (c2, top). PPR did not display differences before (1.125 ± 0.0535) and after HFS (1.132 ± 0.0350, *t_7_* = −1.024, *p* = 0.340, two-tailed, paired *t*-test; c3).

In our earlier findings, we documented that Cdk5 inhibition facilitates the induction of LTP through the D1 receptor signaling pathway in striatal slices from control mice (Miranda-Barrientos et al., 2014). To investigate whether activation of D1 receptors was implicated in the changes of EPSC amplitudes observed in the presence of roscovitine in neurons from 3-NP-treated animals, we conducted recordings on these neurons in the presence of a D1 receptor antagonist SCH23390 (1 μM) and roscovitine (Figure 4A).

In one set of cells (3 of 9), the D1 inhibitor SCH23390 impeded the generation of any type of plasticity in the 3-NP slices in the presence of roscovitine (Figure 4A, a1,a2). However, the D1 antagonist did not block LTP induction in six of nine cells from 3-NP-treated animals (Figure 4B, b1,b3). The PPR was not significantly different in any of the two sets of cells, where no plasticity was developed (Figure 4A, a3) or where LTP was not prevented by SCH23390 suggestive of postsynaptic changes (Figure 4B, b3).

In particular, the administration of the PKA inhibitor H89 (5 μM) to the recording bath blocked the induction of any type of plasticity in the presence of roscovitine in eight of eight MSN cells recorded from the 3-NP-treated animal tissue (Figure 4C, c1,c2). No changes in the PPR were observed. The block of synaptic plasticity in the presence of H89 + roscovitine in 3-NP-treated neurons suggests that PKA signaling is involved in the induction of LTP generated when the Cdk5 is inhibited by roscovitine (Figure 4C).

### 3.5 DARPP-32 phosphorylation state in the 3-NP treatment

Cdk5 phosphorylates DARPP-32 phosphoprotein on its residue Thr-75 (Bibb et al., 1999). Given that the number of positive neurons for Cdk5 increased in tissue slices from 3-NP-treated mice, we wanted to determine whether the changes observed in plasticity were due to changes in the phosphorylation state of DARPP-32. Immunoblot assays were performed to measure total-DARPP-32, as well as the Cdk5 phosphorylation site DARPP-32^Thr75^ and the PKA phosphorylation site DARPP-32^Thr34^ (Hemmings et al., 1984; Snyder et al., 1992) in slices from control and 3-NP-treated mice. These experiments were conducted in the presence of bicuculline, roscovitine, and roscovitine + nifedipine to block other sources of calcium that could activate Cdk5 due to membrane depolarization. Data were expressed as p-DARPP-32/total DARPP-32 and normalized to control protein level.

**Figure 5:**
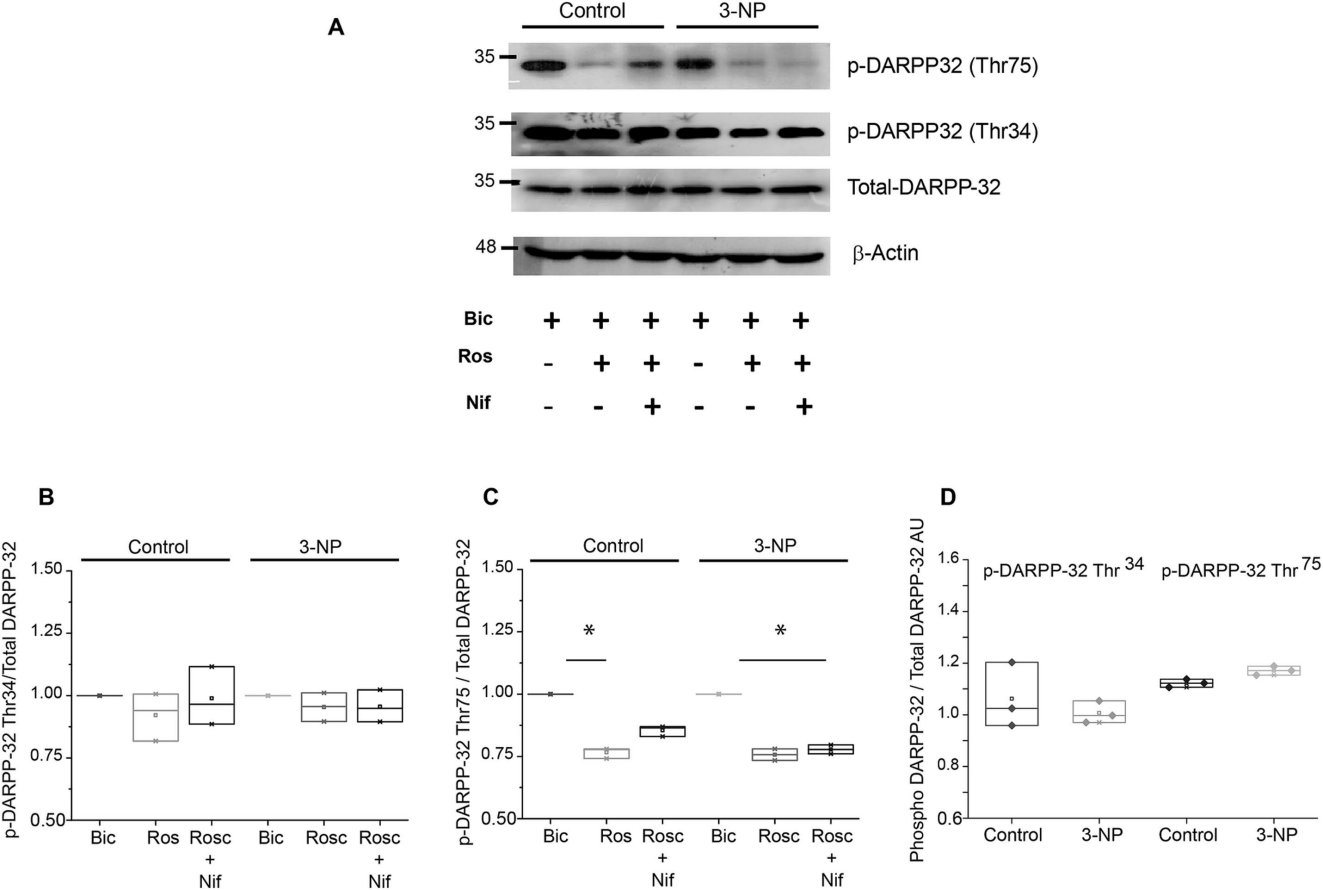
DARPP-32 phosphorylation levels. **(A)** Immunoblot of p-DARPP-32^Thr34^, pDARPP-32^Thr75^, total DARPP-32 protein expression in striatal lysates from five mice treated with 3-NP (15 mg/kg, 5 days) in the presence of bicuculline (control), roscovitine (20 μM), or nifedipine (5 μM). **(B)** Relative p-DARPP-32^Thr34^ levels did not exhibit modifications under the presence of the Cdk5 or L-type Ca^2+^ channel inhibitors (*H_2_* = 0.828, *p* = 0.721, Kruskal–Wallis, one-way ANOVA). **(C)** Relative p-DARPP-32^Thr75^ level significantly decreases in the presence of roscovitine in both control slices (*H_2_* = 7.448, *p* = 0.004, Kruskal–Wallis, one-way ANOVA) and slices of 3-NP-treated animals (*H_2_* = 6.161, *p* = 0.025, Kruskal–Wallis, one-way ANOVA). **(D)** Comparison of p-DARPP-32^Thr34^ and pDARPP-32^Thr75^ levels (arbitrary units) shows that 3-NP treatment did not affect their protein levels compared to the control (*U* = 4, *p* = 1; *U* = 4, *p* = 0.100, respectively, Mann–Whitney rank sum test). *p < 0.05.

The phosphorylation state of DARPP-32 ^Thr34^ in the presence of roscovitine was not significantly affected (Figures 5A,B); however, the level of p-DARPP-32^Thr75^ was significantly reduced in the presence of roscovitine and in the presence of roscovitine + nifedipine in both control and 3-NP-treated mice slices (Figures 5A,C).

In the analysis of the relative expression of p-DARPP-32^Thr34^ and p-DARPP-32^Thr75^ under basal conditions, we observed a non-significant decrease in p-DARPP-32^Thr34^ and there was a non-significant increase in p-DARPP-32^Thr75^ in tissue from the 3-NP-treated group compared to the control group (Figure 5D).

## 4 Discussion

Cdk5 plays a crucial role in various neurological processes, including brain development, dopamine signaling, and synaptic functions within the striatum (Bibb et al., 1999; Hawasli and Bibb, 2007; Cheung and Ip, 2012; Liu et al., 2016). This study seeks to investigate the effect of 3-NP treatment on Cdk5 expression and the resultant alterations in striatal synaptic plasticity. For that purpose, we utilized a 3-NP-induced model designed to simulate the early stages of Huntington’s disease and assessed both the initial damage induced by 3-NP and the role of Cdk5 in disrupting striatal synaptic plasticity. To inhibit Cdk5, we chose roscovitine, a specific cyclin inhibitor that in post-mitotic neurons predominantly targets Cdk5.

### 4.1 Cdk5 immunostaining increases with 3-NP treatment

*In vivo* mice treatment with 3-NP induced an increase in Cdk5-positive staining in cells within the striatum. This immunostaining increase was specifically observed in the soma of striatal cells, compared to control mice. Cdk5 is known to localize at various cellular compartments, including the axon, membrane-associated regions with activator proteins, and the cytoplasm, particularly during heightened activity. This variability in localization could explain the increased staining observed in the soma of neurons from 3-NP-treated mice, without changing significantly protein levels. Previously, it has been reported that neuronal activity modifies the activity of Cdk5 and its cellular localization (Tomizawa et al., 2000). Previous experiments involving the incubation of slices with 3-NP (100 μM) for 3 h have also shown an increase in Cdk5 (Napolitano et al., 2004).

The overexpression and/or overactivation of Cdk5 has been linked to neurodegeneration in HD (Crespo-Biel et al., 2007; Paoletti et al., 2008). The increased Cdk5 level results in the hyperphosphorylation of multiple substrates, regulating neurotransmission, cell trafficking, and cell survival (Cheung and Ip, 2012; Sheng et al., 2015; Liu et al., 2016). However, a Cdk5 increase can also have a homeostatic function on neuronal activity (Sheng et al., 2015; Pao and Tsai, 2021).

### 4.2 Altered striatal plasticity with 3-NP treatment

In cells from the control tissue, only LTD was observed after HFS, while neurons from 3-NP treatment modified striatal plasticity. The cells from 3-NP-treated mice exhibited both LTD and LTP, with the magnitude of LTD being smaller than that observed in the slices from the control mice. The 3-NP treatment also favored the development of LTP in a few cells. Earlier reports have shown that both *in vitro* and *in vivo* administration of 3-NP in rats reduces LTD and induces LTP in field recordings, which supports our findings (Calabresi et al., 2001; Dalbem et al., 2005; Picconi et al., 2006).

### 4.3 Inhibition of Cdk5 with roscovitine affects striatal plasticity in 3-NP-treated mice

The presence of roscovitine resulted in a discrete LTD or LTP in corticostriatal synapses. These effects on striatal plasticity suggest that Cdk5 plays a role in modulating synaptic plasticity favoring LTD. The increase in Cdk5-positive cell staining may indicate a contribution of Cdk5, in part, to the changes in plasticity observed after 3-NP treatment. This is supported by the effects of the Cdk5 inhibitor roscovitine administration, which reduces the strength of LTD in most of the cells and facilitates an LTP with greater amplitude than that observed in 3-NP tissue slices alone in some cells.

### 4.4 Involvement of PKA signaling pathway in roscovitine-induced plasticity

The D1 receptor signaling pathway was implicated in the plasticity induced in only a subset of the recorded cells from 3-NP-treated mice. The addition of the D1 antagonist SCH23390, along with roscovitine, inhibited LTD expression in these cells but not in others that continued exhibiting LTP despite the presence of SCH23390 (Figure 4). Nonetheless, the inhibition of PKA with H89 in the presence of roscovitine blocked the induction of plasticity in all cells, indicating the involvement of PKA signaling in the induction of striatal plasticity generated in neurons from 3-NP treatment. H89 blocked plasticity in all cells because, in the striatum, PKA can be activated by the stimulation of D1 or A2a receptors in the direct and indirect pathways, respectively (Nishi et al., 2011; Ma et al., 2022). While the present study did not specifically evaluate A2a receptors, cells lacking D1/PKA signaling activation may engage in PKA signaling through the activation of A2a receptors.

In the striatum, the expression of LTD and LTP at corticostriatal synapses is known to be finely regulated by dopamine levels. Low levels of dopamine tend to facilitate LTD induction, while elevated dopamine levels favor LTP (Lovinger, 2010). Although both forms of synaptic plasticity can be induced (Lovinger, 2010) and are involved in the control of learned motor programs (Di Filippo et al., 2007), certain forms of LTP are linked to excitotoxic processes or pathological LTP (Centonze et al., 2001).

Cdk5 inhibitors have been shown to enhance dopaminergic transmission (Chergui et al., 2004), because the activations of Cdk5 reduces PKA phosphorylation of DARPP-32 in Thr-34, which is associated with striatal LTP generation (Miranda-Barrientos et al., 2014; Hernandez et al., 2016). Phosphorylation of DARPP-32 in its Thr75 residue by Cdk5 inhibits PKA signaling (Bibb et al., 1999), suggesting that under normal conditions, Cdk5 tends to promote the expression of LTD by preventing PKA activation. Our previous study demonstrated that Cdk5 inhibition facilitates LTP development (Miranda-Barrientos et al., 2014). Consequently, an increase in Cdk5 following 3-NP treatment may trigger a homeostatic mechanism aimed at preventing pathological LTP induction or changes in neuronal activity. It has been noted that Cdk5 plays a crucial role in synaptic scaling, enabling neurons to adapt to changes in activity levels (Seeburg et al., 2008). This potential regulatory response is crucial, especially considering the associated excitotoxicity stemming from altered striatal dopamine levels and a depolarized membrane potential (Zeron et al., 2002). Our 3-NP model imitates changes in the early stages of striatal degeneration; therefore, it is possible that Cdk5 activation prevents DARPP-32^Thr34^ phosphorylation by PKA. To test this idea, we evaluated the phosphorylation state of DARPP-32.

### 4.5 DARPP-32 phosphorylation state in 3-NP treatment

The phosphorylation state of DARPP-32^Thr75^ exhibited a notable reduction in the presence of both roscovitine and in the presence of roscovitine + nifedipine, with no concurrent alteration in DARPP-32^Thr34^. The depolarization of MSN cell membranes by 3-NP may induce Ca^2+^ influx, potentially activating Cdk5. To discern the role of Ca^2+^ entry through L-type channels, nifedipine was employed to eliminate Ca^2+^ influx and its potential contribution to DARPP-32^Thr75^ phosphorylation (Loya-López et al., 2020), but no further inhibition of phosphorylation was observed. Nonetheless, reduced phosphorylation of DARPP-32^Thr75^ with Cdk5 inhibition may leave the PKA/DARPP-32^Thr34^ pathway activated, thereby mitigating LTD and favoring LTP.

Concomitant with the lack of a significant increase in the protein level of Cdk5, protein expression analysis revealed a non-significant increase in the basal phosphorylation of DARPP-32^Thr75^, a site phosphorylated by Cdk5 (Bibb et al., 1999). Additionally, the PKA phosphorylation site, DARPP-32^Thr34^, exhibited a non-significant decrease.

In a previous study, acute administration of a higher dose of 3-NP led to DARPP-32^Thr-75^ upregulation and DARPP-32^Thr34^ downregulation (Napolitano et al., 2004). In our case, the administration of 3-NP in low doses *in vivo* for 5 days, mirroring the initial stages of neurodegeneration, did not significantly alter the phosphorylation status of DARPP-32^Thr34^ and DARPP-32^Thr7^5. Nevertheless, the observed tendency to increase DARPP-32^Thr75^ phosphorylation and decrease DARPP-32^Thr34^ phosphorylation suggests a compensatory mechanism to prevent corticostriatal LTP (Miranda-Barrientos et al., 2014). This mechanism may aim to mitigate the risk of excitotoxicity arising from NMDA receptor activation by mitochondrial dysfunction (Lu et al., 2015) and confers a neuroprotective role for Cdk5 in early neurodegeneration (Pao and Tsai, 2021).

## 5 Conclusion

Our results reveal that, in a pharmacological model induced by 3-NP treatment, which simulates earlier stages of Huntington’s disease (HD), Cdk5 may play a role in attenuating signaling pathways, influencing neurons to enhance their activity. This finding points to a potential involvement of Cdk5 in modulating neuronal responses to neurodegenerative insults and may have implications for potential therapeutic interventions targeting these pathways in neurodegenerative disorders.

It is important to note that roscovitine may affect other cyclin-dependent kinases in addition to Cdk5. While its primary target in neurons is Cdk5, future experiments should investigate more selective Cdk5 inhibitors to assess their roles in both early and late neurodegeneration.

## Data Availability

The data supporting the findings of this study will be made available from the corresponding author upon reasonable request.
