# Supplementary figures and images for: Exploring the role of Cdk5 on striatal synaptic plasticity in a 3-NP-induced model of early stages of Huntington’s disease
Source: Front Mol Neurosci. 2024 Nov 6;17:1362365. doi: 10.3389/fnmol.2024.1362365 (PMC11576431; doi:10.3389/fnmol.2024.1362365)

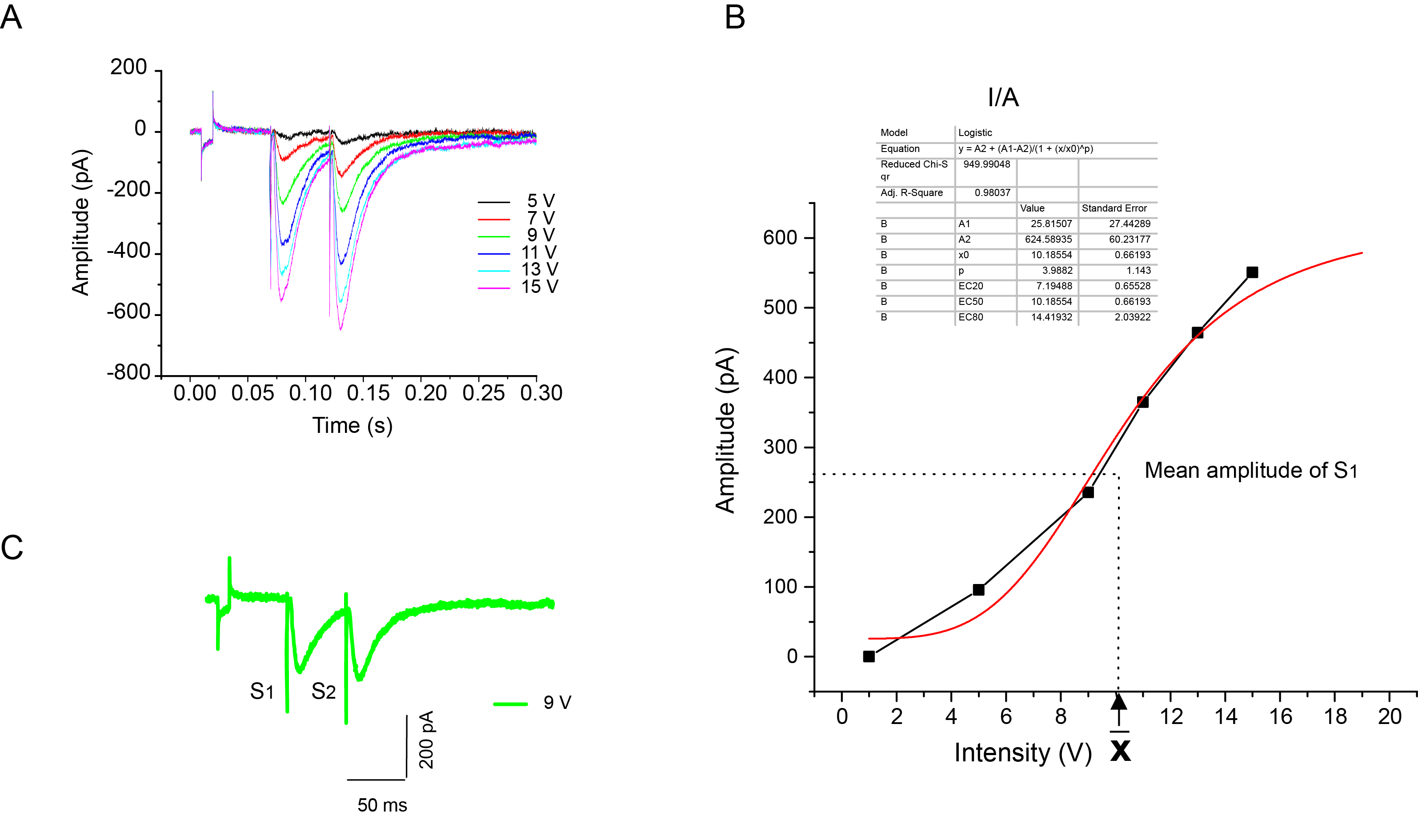

Supplement: Supplementary file 1 [file Image_1.TIF]
